# Obesity and Coronary Artery Disease: An Updated Systematic Review 2022

**DOI:** 10.7759/cureus.29480

**Published:** 2022-09-23

**Authors:** Mohana Priya Manoharan, Rabab Raja, Aneeque Jamil, Denise Csendes, Sai Dheeraj Gutlapalli, Keerthana Prakash, Kiran Maee Swarnakari, Meena Bai, Darshi M Desai, Aditya Desai, Sai Sri Penumetcha

**Affiliations:** 1 Internal Medicine, California Institute of Behavioral Neurosciences & Psychology, Fairfield, USA; 2 Medicine, California Institute of Behavioral Neurosciences & Psychology, Fairfield, USA; 3 Division of Research & Academic Affairs, California Institute of Behavioral Neurosciences & Psychology, Fairfield, USA; 4 Internal Medicine Clinical Research, California Institute of Behavioral Neurosciences & Psychology, Fairfield, USA; 5 Internal Medicine, University of California Riverside School of Medicine, Riverside, USA; 6 General Medicine, California Institute of Behavioral Neurosciences & Psychology, Fairfield, USA; 7 General Medicine, Chalmeda Anand Rao Institute of Medical Sciences, Karimnagar, IND

**Keywords:** overweight, weight loss, metabolic syndrome, body mass index, adipocytokines, metabolic phenotypes, obesity paradox, metabolically healthy obesity, coronary artery disease, obesity

## Abstract

The primary goal is to identify the pathogenesis of cardiovascular illnesses in obese patients. Articles were extracted using the MeSH search approach from PubMed and Google Scholar databases. Inclusion and exclusion criteria were used, and duplicates were eliminated. Eight publications were finally included in this research study after two authors independently completed the quality check appraisal. Seven observational studies and one narrative review were found in our search. The publications evaluated the risk of coronary artery disease in metabolically healthy obese people with that of unhealthy obese adults and evaluated the effects of adipose tissue-mediated inflammation. Additionally, they offered several explanations for the obesity problem. Studies have indicated that adipocytokines and their pro-inflammatory cytokines have significantly affected the development of cardiovascular disease in obese subjects. The relationship between metabolically unhealthy people with increased risk for coronary artery disease (CAD) is unclear. It has also been shown that metabolically healthy obese persons are still at risk for developing coronary artery disease (CAD), as explained in certain studies in which inflammation plays a vital role in obese people. There hasn't been much data on the advantages of being physically active in overweight people, but obese people have to change their lifestyle as a first measure.

## Introduction and background

Obesity and overweight play a key role in the development of cardiovascular disease or myocardial ischemia, particularly today, as does their relationship to traditional and non-traditional risk factors [1]. In this modern society, a new threat to health is the obesity epidemic [2]. The most common cause of death worldwide is cardiovascular disease [2], and obesity is the most critical factor in the development of coronary heart disease [3]. It is believed that the global spread of unhealthy lifestyle factors such as smoking, overweight, inactivity, and type 2 diabetes mellitus can partially explain this increasing change in the global epidemiological pattern [4]. As known, obesity is one of the most significant threats the world is facing, and it’s increasing progressively over the past decades [4]. The first wave of the obesity pandemic has already occurred in some high-income countries, and the second wave has hit some low- and middle-income countries [4]. More than 25% of the adult population in some middle-income nations, including Mexico, Argentina, Libya, Jordan, Egypt, and South Africa, are reported to be obese today. This is a significant change from only two decades ago [4].

Obesity is the increased storage of body fat and its pathogenesis involving multiple factors [5]. A tool used to assess obesity is the body mass index of ≥30 kg/m2 [4]. This statement has limitations, such as fit people with greater muscle mass can have the same body mass index (BMI) as unfit people with larger fat mass [4]. Other measures of obesity that have been suggested are waist-to-hip ratio, waist-to-height ratio, and waist circumference [4]. The risks of developing CAD increased by 40% with every 10 cm rise in waist circumference, with an odds ratio of 1.04 (95% CI: 1.01-1.07, P = 0.013) for a 1 cm increment [4]. The obesity paradox has been seen in some patient populations, but the pathophysiologic mechanisms causing it are not fully understood [6]. Metabolically healthy obesity (MHO) is one type of obesity without any cardiometabolic risk factors such as hypertension, dyslipidemia, insulin resistance, and type 2 diabetes mellitus [7]. It appears that those who exhibit the metabolically healthy obesity (MHO) phenotype do not have an increased risk of developing atherosclerosis [8].

Certain studies have suggested that metabolically healthy obese will progress to metabolically unhealthy obese [8]. Also, the previous results have concluded that, compared to non-obese people, people with MHO are at increased risk for atherosclerosis [8]. Although previous studies have recommended that obesity increases the risk of coronary artery disease, the connection between body mass index (BMI) and age of presentation of symptomatic coronary artery disease (CAD) has not been well explained [3].

Management has been outlined and found effective for coronary artery disease [8]. However, obese patients exhibit myocardial ischemia even in the absence of occlusion of large coronary arteries [9], which can be due to any alterations in the coronary microcirculation [9].On a generalized note, in subjects with type 2 Diabetes mellitus, the coronary microvascular disease is recognized by small artery vasospasm and microvascular obstruction [9], but in obese people before the development of hyperglycemia, it is quite rare for these morphological changes to be observed in the microvessels [9]. Certain studies have observed that myocardial perfusion is reduced in obese people, and others have stated that myocardial perfusion is not altered in obesity [9]. Reduced myocardial perfusion is a claim that may be caused by coronary microvascular capillary’s impaired ability to act as vasodilators, which is a result of some significant cardiovascular risk factors that contribute to the pathogenesis of obesity [9].

The main objective is to conduct a systematic review to ascertain the connection between the obesity paradox, how individuals with metabolic syndrome and obesity are more likely to experience cardiac complications like coronary artery disease, the underlying pathophysiology, and the effects of weight loss on obesity.

## Review

Methods

**Figure 1:**
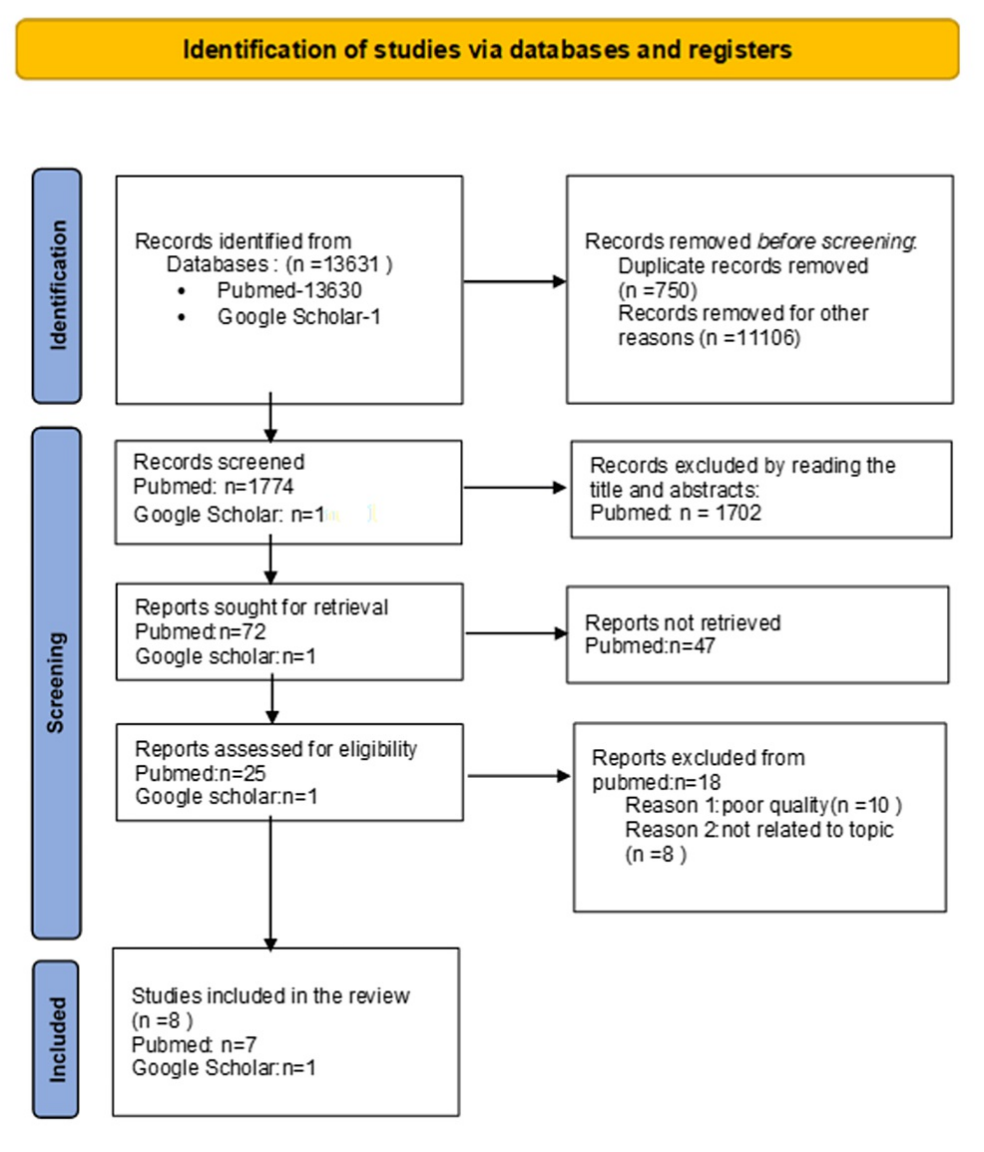
PRISMA Flow Diagram Ref: [10]

We followed the Preferred Reporting Items for Systematic Reviews and Meta-Analyses (PRISMA) guidelines; the MeSH strategy was obtained after finalizing the topic and selecting keywords such as obesity, coronary vascular disease, and myocardial infarction. Articles were accessed from PubMed, and the inclusion and exclusion criteria were applied. The reports relevant to the topic present within the last 10 years and the papers available in the English language alone are selected. Once we obtained the results, duplicates were removed. The documents were also searched manually and obtained from Google Scholar. After this, the articles were screened by topic name and by reading the abstracts. After doing this, full-text papers for the remaining articles were downloaded, and the research articles were further redefined after applying the eligibility criteria (inclusion and exclusion). The next step, quality appraisal or quality check, was done by two authors independently. The quality appraisal tool used was the SANRA checklist; based on this, the articles were included in the final review of the study. Below is Table 1, which shows the search strategy obtained by MeSH. Table 2, which lists the inclusion and exclusion criteria applied, and Figure 1 [10], below explains our search strategy and the literature review process through a PRISMA flow diagram.

**Table 1:** MeSH Search Strategy

SEARCH STRATEGY
Obesity ( "Obesity/complications"[Majr] OR "Obesity/etiology"[Majr] OR "Obesity/pathology"[Majr] OR "Obesity/physiology"[Majr] OR "Obesity/physiopathology"[Majr] ) AND Coronary Heart Disease OR Myocardial Infarction( "Coronary Disease/complications"[Majr] OR "Coronary Disease/etiology"[Majr] OR "Coronary Disease/pathology"[Majr] OR "Coronary Disease/physiology"[Majr] OR "Coronary Disease/physiopathology"[Majr] )

**Table 2:** Applied Inclusion and Exclusion Criteria

Inclusion criteria	Exclusion criteria
Articles associated with the data	Articles not associated with the data
Articles from the last 10 years	Articles more than 10 years
Articles in the English language	Articles that were not in the English language

Results

From a preliminary screening of 13631 papers, eight articles were chosen for participation in the study. Seven of the eight items in this collection were full-text free downloads from PubMed, while one was from Google Scholar. These articles describe how obesity affects coronary artery syndrome and contrast the risks of developing coronary artery disease (CAD) in obese people with and without metabolic abnormalities. The finalized articles are included in Table 3 below, along with their intended audience.

**Table 3:** Study Characteristics

Author	Year of publication	Purpose of study	Intervention studied	Result/ conclusion
Yoosoo Chang [11]	2014	To compare coronary artery calcium scores in metabolically healthy obese individuals and normal-weight individuals.	Coronary artery calcium scores	Metabolically healthy obese subjects have a higher incidence of subclinical coronary artery atherosclerosis when compared to normal-weight individuals.
Zsolt bagi [9]	2014	To find the involvement of adipose tissue mediated inflammatory response	Role of adipokines	The release of adipokines and proinflammatory cytokines from adipose tissue plays an important role in coronary microvascular inflammation
Eiman Jahangir [12]	2014	The relationship between obesity and coronary artery disease	Risk factors of the obesity paradox	Obesity increases the risk of CAD through changes in the endothelial function and other inflammatory responses and also alters the lipid profile
Farzaneh Montazerifar [13]	2012	To evaluate the relationship between serum resistin and leptin levels with obesity and coronary artery disease (CAD)	levels of serum resistin and leptin, C-reactive protein (CRP), lipid profile and cardiac enzyme tests, and calculating of body mass index (BMI), and waist circumference (WC)	Increased levels of leptin and CRP, (p < 0.001), cholesterol (p < 0.05), triglyceride (p < 0.01), and WC (p < 0.05) is found in CAD patients compared to healthy controls.
Fangjian Guo [14]	2016	To assess the cardiometabolic risk in metabolically healthy obesity and unhealthy obesity	Used indices of blood pressure, blood glucose, and blood lipids	MHO subjects have a lower risk of diabetes, stroke, and CHD when compared to unhealthy subjects but diabetes risk is higher when compared with healthy lean people
Diana A. Chirinos [15]	2020	To determine abdominal obesity as a risk factor for CAD	Assess between metabolic syndrome and abdominal obesity	Waist circumference cut-off points determining the risk of CAD
Eva Talavera-Garcia [16]	2016	To determine the influence of isolated overweight and obesity on carotid intima-media thickness and also whether it is associated with metabolic abnormalities	Metabolic phenotypes and carotid artery ultrasound	Metabolically healthy subjects have greater carotid intima-media thickness when compared to metabolically healthy subjects
Tiffany M. Powell-Wiley [17]	2021	To determine the influence of obesity on cardiovascular disease	Invasive and non-invasive diagnostic tests	Further need to evaluate the mechanisms for obesity-related cardiac dysfunction and management options

Discussion

Pathophysiology of Obesity in Coronary Artery Disease (CAD) 

The pathological role and management of atherogenic dyslipidemia in the development of obesity-related coronary artery disease are well known [9]. Adipocytokines have a significant role in the incidence of cardiovascular diseases [13]. The hormones like peptides and other molecules secreted from the adipose tissue likely act as pro-atherogenic markers [13]. Adipokines released from adipocytes consist of adiponectin, leptin, resistin, vascular endothelial growth factor (VEGF), and pro-inflammatory cytokines, such as TNF, IL-1, IL-6, and monocyte chemoattractant protein-1 (MCP-1) [9]. In obesity, the endocrine function of adipocytes is changed, with decreased adiponectin [9] and increased levels of leptin, resistin, IL-6, and tumor necrosis factor (TNF) [9]. During the prolonged follow-up periods, it was observed that patients who had been obese at baseline stayed obese and infrequently turned lean. This was true for both young adults and young-older adults who were obese [14]. The prognosis of CAD can sometimes be evaluated with the serum level of adipokines, which is used in screening, diagnosing, and predicting atherosclerosis [13]. Increased body mass leads to increased metabolic requirements [9]. In "uncomplicated" obesity, enlarged left ventricular mass might be an early adaptation of cardiac function, compensating for the greater hemodynamic and metabolic demand in obesity [9]. Inflammatory processes linked to obesity occur in both vascular and non-vascular tissues, and endothelial cells are activated by the vasoactive chemicals secreted by adipocytes, such as resistin and leptin [13].

Role of Adiponectin, Resistin, and Leptin

In general, adiponectin is found to have a protective effect on the heart and safeguards from ischemia-reperfusion injury via a combination of AMP kinase- and cyclooxygenase-2-dependent mechanisms [9]. In obesity, this effect declines because of low adiponectin levels [9]. Also, it raises nitrous oxide (NO) bioavailability, producing vasodilation [9]. It contributes to the loss of vasodilator properties in obese people with metabolic syndrome [9]. Insulin resistance diabetes develops in obese individuals as a result of low levels of adiponectin, which is the primary consequence of the insulin-sensitizing hormone [9]. Adiponectin has a trimeric, hexameric, and high molecular weight structure in serum and cells. A defect in adiponectin multimerization affects the protein's stability and secretion, which is linked to insulin resistance [9].

The role of an appetite-regulating hormone known as leptin is weight reduction by acting through hypothalamic neurons, activating the catabolic pathway, and inhibiting the anabolic pathway [9]. Therefore, lower leptin levels were associated with increased weight gain in healthy individuals [9]. In contrast, obese people were found to have higher leptin levels denoting leptin resistance rather than inadequate leptin production [9]. Additionally, elevated levels of leptin also modify the vasomotor function in obesity [9]. With leptin, there is greater endothelial NO synthase (eNOS) expression with low intracellular L-arginine levels contributing to the uncoupling of eNOS and production of superoxide and peroxynitrite production [9]. But the overall contribution of adipocyte-derived leptin to coronary artery dysfunction remains unclear [9].

Resistin plays a vital role in metabolic homeostasis and is found to be elevated in obesity [9]. The known potent vasoconstrictor endothelin -1 expression is increased by resistin [9]; glucose and glucocorticoids have a significant role in the induction of resistin, whereas insulin and TNF inhibit the resistin expression [9]; greater plasma resistin levels are interrelated with inflammatory markers such as TNF receptor-2, IL-6, lipoprotein-associated phospholipase A2 and with higher coronary calcium score which measures the severity of coronary sclerosis [9]. In the secretory vesicles found in adipocytes, leptin and resistin are grouped into each secretory vesicle, where its secretion is modulated by the cellular level of cAMP and protein kinase A as well as insulin/glycolytic substrates [9]. In patients with symptomatic coronary artery disease, a correlation was discovered with resistin [9].

Certain studies have also concluded that adipokines such as resistin, leptin, and adiponectin have adverse effects on coronary arteriolar dilation in obesity which in turn is related to the development of coronary artery disease [9]. These effects are supported by the loss of NO and increased reactive oxygen species (ROS) production in the coronary arteries of obese subjects [9]. Obesity is connected with inflammation, evidenced by increased C-Reactive protein (CRP) levels [13]. Studies have proved that inflammation associated with elevated levels of resistin and leptin has a significant role at the beginning of the mechanism of inflammation and leads to the advancement of atherosclerotic disease [13], as a known factor that CRP is an important marker determining the degree of inflammation, this elevated levels of resistin and leptin, in turn, induces the production of CRP in coronary endothelial cells and this CRP promotes vascular thrombosis that might be involved in the acute coronary syndrome pathophysiology process [13]. In that same study, it has shown that leptin and resistin are linked with coronary artery disease regardless of CRP [13].

Cardiometabolic Criteria

Wildman has formulated cardiometabolic abnormalities [16]. Guidelines defined by CDC/AHA contain blood pressure more than 130/85 or currently using any antihypertensive drug, Triglycerides more than or equal to 150mg/dl, fasting blood sugar more than 100 mg/dl [16], or on any anti-diabetic treatment, high-density lipoprotein (HDL) less than 40 mg/dl in men or less than 50 mg/dl in women, or lipid-lowering treatment is used, insulin resistance more than 2.6 and CRP more than 3mg/dl [16].

*Metabolic Phenotypes* 

Metabolically healthy normal weight includes BMI < 25 and two metabolic criteria [16]. Metabolically sick normal weight has BMI <25 and more than or equal to two metabolic measures [16]. Metabolically healthy overweight includes BMI ranging between 25 to 30 and less than two metabolic criteria [16]. Metabolically sick overweight has BMI between 25 to 30 and more than or equal to two metabolic measures [16]. Metabolically healthy obese include a BMI of more than 30 and less than two metabolic criteria, whereas metabolically sick obese includes more than or equal to two metabolic standards and a BMI of more than 30 [16].

Components of Metabolic Syndrome

Obesity and overweight are the major determinants of metabolic syndrome and contribute to the development of cardiovascular diseases [18]. Obesity is also a significant risk factor for both DM and metabolic syndrome, and increased cases of obesity have been seen due to the prevalence of DM [12]. Metabolic syndrome constitutes a combination of anthropometric, hemodynamic, and metabolic alterations [15]. In 2009, a clinical diagnosis of metabolic syndrome was released by a joint interim statement issued by several organizations [15]. This statement compromises a cluster of components such as central obesity, elevated blood pressure and triglycerides, low high-density cholesterol, and altered glucose metabolism [15]. Metabolic syndrome has a stronger association with developing atherosclerotic cardiovascular disease [19] both genetic and acquired factors are involved in the pathogenesis of metabolic syndrome, which in turn leads to the final pathway of inflammation contributing to coronary vascular disease [16]. Weight (kg) divided by height (m2) can be used to calculate BMI [7]. The World Health Organization defined obesity as having a BMI of 30 kg/m2, overweight as having a BMI of 25, and average weight as having a BMI of 18.5 kg/m2 [7]. When the metabolic syndrome is identified early, patients should be actively encouraged to make lifestyle changes, and physicians can take steps to reduce the risk of type 2 diabetes mellitus [15]. The management of patients already diagnosed with T2DM-associated metabolic syndrome is crucial because it helps to overcome the known cardiac complications associated with T2DM [15].

Metabolically Healthy and Unhealthy Obesity 

Metabolically healthy subjects can be stated as not having any metabolic risk factors such as elevated blood pressure, triglycerides, not using any drugs for hypertriglyceridemia, and decreased HDL cholesterol levels [20]. Metabolically unwell people are those who have one or more of the aforementioned risk factors. [20]. Evaluation of metabolic health status has been demonstrated to help predict the result of cardiovascular risk status [14]. Additionally, it has been noted that obese individuals, even those without metabolic syndrome, have a higher risk of myocardial infarction [14]. Modified metabolic syndrome has been predicted based on glycemic statuses, such as self-reported diabetes mellitus (DM), registry-documented diabetes diagnosis, antidiabetic therapy, and/or nonfasting plasma glucose level greater than 200 mg/dl. [14]. In the CARDIA study over 20 years, 67.3% of baseline overweight participants and 17.5% of baseline lean subjects both converted to obesity at the year 20 evaluation [14]. In comparison to younger patients in CARDIA, fewer older patients in ARIC transitioned from being lean to being obese or from being overweight to being obese, suggesting that BMI status was more stable over 10 years in subjects in their sixth and seventh decade than it was in middle-aged adults [14]. The rate of glucose tolerance alone in a metabolically unhealthy subject, apart from other components of metabolic syndrome, has a greater effect on CAD [20]. Metabolically healthy obese people seem to have higher coronary artery calcium scores [11]. Also, obese people might have fibrinolysis impaired and increased hypercoagulability [11] because atherogenesis is mediated by interleukin - 6 and tumor necrosis factor-alpha released from adipose tissue [11]. Studies have concluded that metabolically unhealthy subjects have an increased risk for CAD compared to people with metabolically healthy obesity [14].

On the other hand, metabolically healthy obese (MHO) people have an increased risk of becoming ‘metabolically unhealthy obese [21]. Significantly, metabolically healthy individuals have reduced quality of life because of the prevalence of other obesity-related comorbidities such as psychological abnormalities, osteoarthritis, respiratory distress, gynecologic abnormalities, and skin problems [21]. When compared to metabolically unhealthy obese people, metabolically healthy obese subjects have more abdominal subcutaneous adipose tissue, lower visceral fat mass, and less fat accumulation in liver and skeletal muscle with more adipocytes, less macrophage infiltration, and inflammation which, in turn, concludes that MHO subjects having good inflammatory profile [21].

The Obesity Paradox

As discussed above from various studies, although there is a greater risk of acquiring CVD in obese people, recently, it has been stated that once CVD gets established in overweight or obese individuals [12], they have lower mortality when compared with normal-weight people, which is termed as ‘obesity paradox’ [12]. This paradox has been explained in many cardiovascular diseases, such as CAD, atrial fibrillation, and heart failure [12]. Mortality rates for overweight and obese men were comparable to those of the highly fit normal-weight reference group [12]. Patients with high levels of fitness typically have lower mortality rates than those with lower levels, and adding fitness to other conventional risk factors seems to reduce cardiovascular mortality [12]. Defining obesity based on BMI might be one of the reasons for this paradox, as it does not take into consideration lean mass [22]. It has been proposed that before a CVD event, positive caloric balance causing adiposity leads to pathologic changes in the adipose tissue causing metabolic diseases [12]. Alternatively, the improved clinical outcome has been observed with negative caloric balance as it may occur during a CV event where adipose tissue responds with enhanced function [12]. Increased muscle strength in obesity has also been associated with a better prognosis [12].In this evaluation of the obesity paradox, BMI is a protective factor in both lower and higher CRP groups [23]. In obese people with CAD, endogenous regenerative capacity can be measured by using cell progenitor counts, which also explains this obesity paradox [6]. The relation of obesity with adverse outcomes in CAD is indirectly proportional to those with a preserved endogenous regenerative capacity which is higher cell progenitor cell counts [6]. The analysis of this paradoxical association finding remains unclear, and also, many theories have been proposed to support this obesity paradox [22].

Obesity as a Risk Factor for Coronary Artery Disease (CAD) 

In the past two decades, the global epidemic affecting both children and adult populations has been obesity [24]. The adverse effects of obesity are due to pathogenesis involving psychosocial, biological, environmental, and socioeconomic factors [17]. The association between obesity and cardiovascular diseases has been exclusively studied, but the particular question is still not understood and remains complex [24]. Obesity with comorbidities such as hypertension, dyslipidemia, and glucose intolerance increases the risk of coronary vascular diseases [24]. When compared to BMI, the measurement of waist circumference was also found to be a marker for cardiometabolic risk [25]. Obesity can cause CAD, and researchers are looking into the genes that contribute to the development of obesity to see if there is a link to CAD development [5]. one of the molecular mechanisms responsible for early life obesity is an epigenetic modification of genes through methylation, histone modification, chromatin remodeling, and non-coding RNA alterations [26]. This epigenetic modification increases the risk of getting adult obesity and which can be transmitted to future generations, thereby responsible for the obesity epidemic [26]. While certain types of obesity are brought on by solitary mutations, the majority of cases are polygenic and come about as a result of a complicated interaction between the environment and the genotype [5]. The degree of calcification in the coronary arteries can be measured using CT, known as coronary artery calcium scores (CACS) [27]. This CACS score is one of the indicators of atherosclerosis [27], a positive association between high BMI and risk of CAD has been demonstrated, which showed that every 1kg/m2 increase in BMI led to a 5%-7% increase in the incidence of CAD across all BMI categories [28]. For very muscular people, BMI may sometimes overestimate body fat, and for those who have lean muscle mass, BMI may underestimate body fat [28]. A BMI of more than 30kg/m2 is used to measure general adiposity, and a waist-hip ratio of more than 0.90 for males and 0.85 for males is used to measure central adiposity and its association with CAD [28]. Another study that used G-estimation for the assessment of obesity and CHD has concluded that a shorter survival rate for CHD is mainly linked with greater levels of abdominal obesity, either predicted through waist circumference or waist-to-hip ratio [29]. In G-estimation, three criteria were used to calculate the impact of obesity on CHD and compare it to accelerated failure time models. All indices of obesity were associated with an elevated risk of CHD in the first model that was adjusted for baseline variables while removing metabolic mediators of obesity [29]. Additional adjustments in the second model to account for metabolic mediators and the third model to account for time-varying factors revealed minimal hazard ratios [29]. Based on waist circumference and waist-to-hip ratio, respectively, the hazard ratios derived by G-estimation for general obesity were 1.15 (95%CI: 0.83-1.47), 1.65 (95%CI: 1.35-1.92), and 1.38 (95%CI: 1.13-1.99) for abdominal obesity, indicating that abdominal obesity enhanced the risk of coronary heart disease [29].

Methods Used for Assessment of Coronary Artery Disease (CAD) in Obesity

ECG is extensively available and cheap, but its sensitivity and specificity are found to be low [17]. The ECG findings for obese people are displacement of the heart with an elevation of the diaphragm in the supine position, greater cardiac workload, and the distance between the heart and the recording electrodes are increased [17]. Clinically significant changes in ECG include increased heart rate, increased QRS and QT interval [17]. In obese people, treadmill stress test performance is limited, and their aerobic capacity is lowered due to pulmonary dysfunction, orthopedic limitations and left ventricular diastolic dysfunction [17]. These obese people may sometimes stop the stress test because of fatigue, leg pain and dyspnea [17], and the systolic and diastolic blood pressure are elevated while performing the stress test [17]. Single-photon emission CT is used in patients with lower-weight individuals, and it is usually avoided in patients whose BMI is more than 35 kg/m2 [17]. To generate better images in obese subjects, sometimes technetium sestamibi is used as a marker [17]. Because of the limitation in this single photon emission CT, an alternate imaging modality used for myocardial ischemia is positron emission tomography [17]. The assessment of perfusion defects in left ventricular ejection fraction and the detection of scar can be obtained using a technique called stress cardiac MRI with the use of gadolinium [17]. PET rubidium is faster than single-photon emission CT [17]. And has advantages like good quality images, less exposure to radiation and better diagnostic precision but the availability of these tests is limited [17]. In all obese individuals, PET is linked to fewer cardiac fatalities if myocardial perfusion imaging results are normal. [17]. The quantification of both coronary and non-coronary calcified plaque can be evaluated by CT coronary angiography [17]. Plaque characterization and quantification and luminal stenosis evaluation can be done using this CT coronary angiography, whereas CAC allows only risk stratification and assessment of plaque burden [17]. The two invasive evaluations are coronary angiography and invasive coronary ultrasound [17].

Impact on Weight Loss

With regular physical activity and aerobic exercise, the risk factors of CAD can be moderately reduced, evidenced by low body fat and body mass, low blood pressure, low triglycerides, and increased high-density cholesterol [30]. Improves insulin sensitivity and endothelial function regardless of weight loss [17]. The general idea of this weight loss management is to reduce body weight so as to prevent further weight gain and maintain a lower body weight [17]. The overweight or obese individuals with weight loss targets of 5% to 10% have been shown to significantly improve the health outcome of obesity-associated complications [31]. Losing weight can aid in the prevention of T2D in people who are obese and have prediabetes, and it has a favorable long-term effect on cardiovascular mortality [31]. The efficacy of weight loss treatment can be determined by measuring BMI and WC [17]. Also, there is no evidence that studies demonstrate a reduction of coronary vascular disease or mortality with lifestyle modification such as exercise alone [17]. The degree of weight loss obtained (5-10 kg with medical weight loss versus 10-40 kg with surgery) and the reduction in risk factors seen with bariatric surgery are likely the causes of the discrepancies between the outcomes of weight loss trials using medical and surgical methods [17]. But what is certain is that for obese patients with CAD, fitness seems to improve prognosis, and therefore, physical activity and exercise training are recommended as it is accompanied by purposeful weight loss [12].

Limitations

There are several limitations included in this study. The articles included are mainly observational studies, as it does not mean to prove causation. Since the sample size varies in each study, the outcomes that have been discussed cannot be applied to all age groups. This study was also conducted with articles taken from the English language, so the conclusions in other language articles were not known.

## Conclusions

With a sedentary lifestyle becoming more prevalent nowadays, the relationship between obesity and the development of coronary artery disease has been explained by the pathophysiology involved. Adipokines from adipose tissue, such as resistin, leptin, adiponectin, and TNF, play a vital role in the inflammation process, which is the main reason for the development of atherosclerosis. Obesity is a significant component of metabolic syndrome. With six types of metabolic phenotypes present, a greater risk of CAD is associated with metabolically unhealthy people when compared to metabolically healthy subjects. Additionally, a paradox exists that says obese people with CAD are associated with decreased mortality. This research has also yet again shown that inflammation is the major culprit, which plays an important role in the development of coronary artery disease. The articles that were shortlisted and included in this study have a common association that inflammation and further endothelial damage caused by the release of pro-inflammatory cytokines play a vital role in the development of coronary artery disease in obese people, and further, these articles show that metabolically unhealthy obese people have a greater risk of coronary artery disease when compared to metabolically healthy obese people. Additionally, it has been proven that obese people can benefit from losing weight. Even though so many studies have supported the notion put forth above, the relationship between obesity and CAD is still not clear. Future research should take this into account, and more randomized controlled trials should be conducted because the majority of studies currently available are only observational studies.
